# Prevalence of Dental Trauma and Receipt of Its Treatment among Male School Children in the Eastern Province of Saudi Arabia

**DOI:** 10.1155/2020/7321873

**Published:** 2020-09-01

**Authors:** Asim Al-Ansari, Muhammad Nazir

**Affiliations:** Department of Preventive Dental Sciences, College of Dentistry, Imam Abdulrahman Bin Faisal University, P. O. Box 1982, Dammam 31441, Saudi Arabia

## Abstract

**Background:**

Dental trauma is a common dental public health problem, and it affects 20% to 30% of permanent dentition worldwide.

**Objective:**

To evaluate self-reported dental trauma to permanent anterior teeth and the receipt of dental treatment among male school children.

**Materials and Methods:**

This cross-sectional study included grade 7 to 9 school children in Dammam/Al-Khobar, the Eastern Province of Saudi Arabia. The participants responded to a pilot-tested self-completion questionnaire which contained questions about experience, types, place, and reasons for dental trauma and the receipt of dental treatment. Bivariate and multiple logistic regression analyses were performed.

**Results:**

There were 258 students in the study with a mean age of 14.29 ± 1.11 years. Dental trauma was experienced by 39.5% of the participants. Tooth fracture (22.7%) was the most common type of dental trauma followed by tooth displacement (8.7%) and complete tooth removal (8%). The most common reason of dental trauma included fall (9.3%) and accidental hit by some objects (8.9). Home (19.8%), school (5%), and playground (4.2%) were reported as common places of dental trauma. Dental treatment was received by 20.5% of the samples. Most participants visited a dental clinic (10.8%) and used self-care at home (7.2%) after dental trauma. Nearly 4.7% of the participants received dental treatment immediately, 5% on the next day, and 2.7% after a month. Multiple logistic regression analyses showed a significant association of monthly family income (odds ratio = 0.44) with dental trauma (*P* = 0.008).

**Conclusion:**

Dental trauma was highly prevalent among school children; however, few of them received care/dental treatment. Participants frequently experienced dental trauma due to a fall in their homes. Preventive measures should be taken to prevent dental trauma, reduce its burden, and improve quality of life.

## 1. Introduction

Traumatic dental injuries occur in preschool children, adolescents, and adults, and they affect about 20–30% of permanent dentition worldwide [1]. Nearly 80% of dental trauma occurs under the age of 20 years making childhood and adolescence highly vulnerable periods for traumatic dental injuries [2]. Depending upon the severity, type, and duration of dental trauma, various complications such as the fracture of the crown, discoloration of the tooth, necrosis of the pulp, apical periodontitis, root resorption, and fistulas can occur [3]. Evidence shows that children who experienced trauma to their anterior teeth were more likely to avoid smiling, laughing, and were more concerned with their personality than children without dental trauma [4].

Similarly, children with untreated dental trauma face embarrassment and social isolation and known to have a poor oral health-related quality of life [5]. It is documented that adolescents with fractured teeth have an impact on their daily living 20 times more than those without dental trauma [6]. Likewise, untreated dental trauma in adolescents negatively impacts eating and smiling [7]. Besides the functional, physical, and psychological impacts of dental trauma, treating dental trauma can be expensive. For example, the results of a study showed that the annual treatment cost of dental injuries was between US $ 3.3 and 4.4 million per one million individuals in Sweden [8]. Another study also reported that the treatment cost of dental injuries ranged from US $ 2 to 5 million per one million people per year in Denmark [9].

The maxillary central incisors are the most commonly affected teeth with dental trauma, and the fracture of tooth enamel is the most frequent type of dental injury followed by enamel dentine fracture [10, 11]. Various factors such as age, socioeconomic status, and environmental influences play an important role in the etiology of dental trauma. Researchers have shown that adolescents who experienced adverse life events such as low socioeconomic status and poor environmental conditions were more likely to experience dental trauma than those who had positive and economically favorable life experiences [12, 13].

In Riyadh, Saudi Arabia, Al-Majed et al. reported that 34% of boys aged 12–14 years had dental trauma [14]. Also, Al-Majed reported that the prevalence of dental trauma was 31.4% among 12–15-year-old school girls [15]. Al-Malik conducted a study in Jeddah, Saudi Arabia, and observed the highest number of dental injuries in 9–11-year-old children [16]. However, the literature study is limited to dental trauma among school children in the Eastern Province of Saudi Arabia. Therefore, it was important to investigate various aspects related to dental trauma and the receipt of its treatment. Evaluation of factors associated with the occurrence of dental trauma and receipt of treatment can guide the development of effective preventive strategies. The objective of the study was to evaluate self-reported dental trauma among male school children in the Eastern province of Saudi Arabia. The study also assessed the type and place of dental trauma, reasons for dental trauma, and receipt of dental treatment.

## 2. Materials and Methods

This cross-sectional study included middle school children (aged 12 to 15 years) from Al-Khobar, Dammam, and Dhahran, Eastern Province of Saudi Arabia. A sample of 318 participants was calculated based on 95% confidence level, response proportion (30%), the margin of error (5%), and population (*N* = 20,000). The permission to conduct the study was obtained from the school authorities. Any child with erupted permanent anterior teeth was eligible to participate in the study. Children with cleft lip and cleft palate, dental developmental abnormalities, unerupted permanent anterior teeth, and those who lost their anterior teeth due to dental caries or other than dental trauma were excluded from the study. The purpose, objective, and details of the present study were provided to the parents/legal guardians of children in the consent form. The children were included in the study if their parents/legal guardians provided written consent, and they showed willingness to participate in the study. A convenience sampling method was used for the selection of participants. The study was approved by the institutional review board by Imam Abdulrahman Bin Faisal University, Dammam. Ethical guidelines of the Declaration of Helsinki were followed during the study.

The first version of the questionnaire was developed in the English language [14, 15, 17–20]. This was then circulated among two faculty members in the College of Dentistry to assess the appropriateness of its content and structure. This review process helped to confirm the content validity of the questionnaire. The final version of the questionnaire was then translated into the Arabic language. Prior to data collection, pilot testing of the questionnaire was carried out on 30 children to assess the comprehension, clarity, and format of the questions and the duration to complete the survey.

The questionnaire consisted of two parts: the first part included demographic information, and the second part contained questions specific to dental trauma. Demographic data included age, paternal education, monthly family income, and previous academic grades. Dental trauma questions were about the experience of dental trauma to permanent anterior teeth, types and place of dental trauma, reasons for dental trauma, and the receipt and timing of dental treatment.

The study participants (*N* = 318) were provided with a paper-based self-administrated questionnaire in their classrooms. Their queries about questionnaire items and the study were addressed during the questionnaire administration. The questionnaires were collected immediately after the session which was on average about 30 minutes long. The questionnaires with incomplete and/or illegible responses were discarded (*N* = 60).

Statistical Package for Social Sciences (SPSS) for Windows, version 22 (IBM Corp., Armonk, N.Y., USA), was used to perform descriptive and inferential statistical analyses. Frequencies and proportions of categorical variables such as class, paternal education, monthly family income, and previous academic grades were calculated. Means and standard deviations of the continuous variable such as age were calculated. Chi-square test was performed to determine the association between sociodemographic factors and dental trauma and receipt of dental treatment. Multiple logistic regression analyses were used to assess the association between sociodemographic factors and dental trauma and receipt of dental treatment after controlling for confounding factors. Statistical significance was set at a *P* value of less than 0.05.

## 3. Results

Two-hundred and fifty-eight (258) male students from grade 7–9 participated in the study with the mean age of 14.29 ± 1.11 years. About half of the participants (51.2%) belonged to middle-income families (monthly family income equal to 10,000–20,000 SAR; one 1000 SAR is equivalent to $US 267). Having dental trauma was reported by 39.5% of the participants. However, 20.5% of the participants received dental treatment (Table 1).

**Figure 1:**
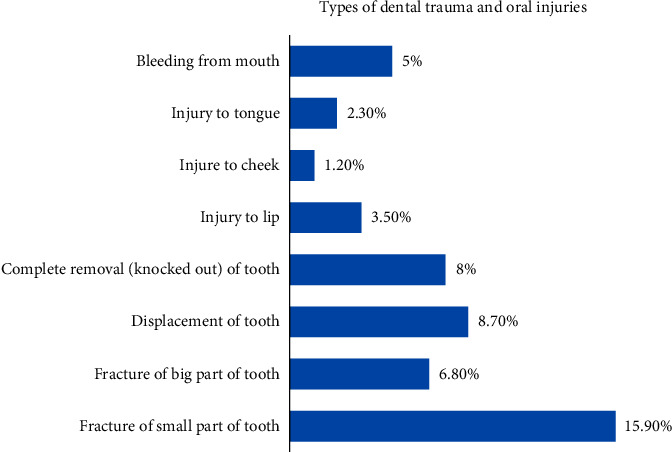
Distribution of participants' responses about the types of dental trauma and oral injuries.

Regarding the types of dental trauma, participants reported tooth fracture (22.7%), tooth displacement (8.7%), and complete tooth removal (8%). Oral injuries included bleeding from mouth (5%), injury to lips (3.5%), injury to tongue (2.3%), and injury to cheek (1.2%) (Figure 1).

**Figure 2:**
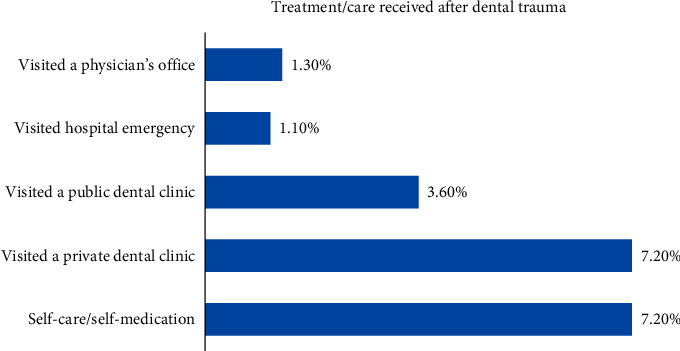
Distribution of participants' responses about dental treatment/care received after trauma.

Fall on the ground was the most common reason for dental trauma (9.3%) followed by an accidental hit by some objects (8.9). On the other hand, intentional hitting by someone in fight or violence was the least common (4.2%) reason for dental trauma. Most participants reported home as a place of dental trauma (19.8%) followed by the school (5%) and playground (4.2%). Dental treatment was received immediately by 4.7% and on the next day by 5% of the participants. Nearly 2.7% of the participants received dental treatment after more than a month of dental trauma. Regarding treatment/care received after dental trauma, 10.8% visited a public/private dental clinic and 7.2% used self-care/self-medication. Visiting a physician for dental treatment was reported by 1.3% of the participants (Figure 2).

In bivariate analysis, being a 9^th^ grader was significantly associated with increased likelihood of having dental trauma (OR = 1.69, *P*=0.047). On the other hand, high family income was significantly associated with lower odds (OR = 0.48, *P*=0.004) of having dental trauma. In multivariate logistic regression analysis, high family income remained significantly associated with lower odds of having dental trauma (OR = 0.44, *P*=0.008). Paternal education and the academic score of participants had no significant association with dental trauma in both bivariate and multivariate analyses (Table 2).

Table 3 shows the association of sociodemographic factors with the receipt of treatment for dental trauma. Being a 9^th^ grader (OR = 0.43, *P*=0.045) and high family income (OR = 0.41, *P*=0.005) were significantly associated with lower likelihood of receiving dental treatment for trauma in bivariate analysis. However, high family income remained a significant factor associated with lower odds of receiving dental treatment (OR = 0.41, *P*=0.017) in multivariable logistic regression analysis.

## 4. Discussion

The study identified that 39.5% of adolescents had dental trauma to their permanent anterior teeth. This finding is higher than what was reported in previous studies in Saudi Arabia which showed that the prevalence of dental trauma ranged from 31.4% to 34% in adolescents [14, 15]. Globally, the distribution of dental trauma among adolescents varies in different countries. For instance, dental trauma was reported in 9.1% of Nigerian [21], 10.9%–14.4% of Indian [18, 22], 16.5%–34.79% of Brazilian [11, 23], 35.0% of Thai [24], 36% of Iranian [25], 18.5% of Canadian [26], and 43.8% of British school children [27]. These dental trauma inequalities in different parts of the world can be explained by the differences in study designs, sample size estimates, sampling techniques, inclusion/exclusion criteria, and measurement methods. In addition, variations in behavioral, cultural, and environmental factors in different geographic locations predispose children to varying degrees of dental trauma [10].

In the present study, only 20.5% of the participants received dental care for dental trauma. A previous study of Brazilian school children reported that 27.6% of teeth with dental trauma received dental care [28]. Another study from Brazil showed that 26% of teeth with dental trauma were restored [11]. On the other hand, Hamdan and Rajab observed that only 3.1% of traumatized teeth were treated in Jordanian school children [29]. Al-Majed et al. reported dental treatment in 2.4% of 12–14-year-old boys in Saudi Arabia [14]. Our study also showed that only 10.8% of the participants visited a dental clinic, and 4.7% received dental treatment immediately. In addition to various aspects of poor quality of life, dental trauma can lead to clinical complications such as external root resorption, ankylosis, and pulp necrosis [30]. Nevertheless, a low prevalence of treatment for dental trauma in our study could be related to low priority for oral health, lack of awareness about the availability of dental care, and expensive dental trauma treatment in addition to sociodemographic variables.

Fall was the most common reason for dental trauma in the present study. Several previous studies locally and globally reported similar findings [16, 18, 19, 22, 30, 31]. In addition, accidental hit by an object was the second most common reason for dental trauma in the present study. In agreement with other studies [19, 28, 31], the home was the most frequently reported place of dental trauma in the present study. It is known that there is a significant association between overcrowded household and dental trauma in school children [27]. Therefore, dental trauma in crowded houses may occur due to fall and accidental hitting. These findings highlight the importance of establishing collaboration among different government departments to develop guidelines to prevent falls and dental trauma in households.

The analysis of sociodemographic factors on dental trauma and the receipt of dental treatment was performed in the present study. The multiple logistic regression analysis showed lower odds of dental trauma among children from high-income families than low- and middle-income families. Similarly, Årtun and Al-Azemi demonstrated a reduced risk of dental trauma among adolescents from high-income families in Kuwait [32]. Our study also demonstrated reduced odds of dental trauma among children with high paternal education, though the relationship was not significant. Damé-Teixeira demonstrated significantly greater likelihood of dental trauma to at least one tooth among children from low socioeconomic status [23]. Low and middle socioeconomic status school children most likely live in crowded houses which increase their chances of experiencing dental trauma. Conversely, high parental education results in increased awareness about the prevention of dental trauma and reduced incidence of dental trauma [23, 27]. These explain the reasons behind reduced likelihood of dental trauma among adolescents from high-income and high-educated families in the present study. On the contrary, Hamdan and Rajab showed no significant association between socioeconomic factors and dental trauma [29].

Patient compliance and cooperation, commonly encountered problems during childhood and adolescence, present challenges for the management of dental trauma [2]. The present study demonstrated significantly lower odds of receiving dental treatment for dental trauma among children from high-income families. In addition, children from university-educated families showed reduced chances of dental treatment after trauma. Low risk of dental trauma among adolescents of high socioeconomic status may account for the reduced likelihood of receiving treatment for dental trauma. However, the interplay of behavioral, psychological, environmental, and socioeconomic factors in the etiology of dental trauma should be considered while evaluating the influence of factors on the receipt of dental treatment.

The study evaluated different factors related to dental trauma and the receipt of dental treatment in adolescents and added valuable information to the available body of evidence on this topic. However, there are certain limitations to this study. Despite the measures to obtain valid data in the present survey, self-reported responses are subject to biases such as recall bias and social desirability bias. In addition, clinical examination of dental traumatic injuries provides more accurate information than self-reported data [25]. Furthermore, a cross-sectional study design cannot be used to infer the associations reported in the present study as causal relationships. Children were recruited from public schools in few cities of the Eastern Province, and the conclusions regarding generalizability should be drawn carefully. Large prospective studies are required to understand various epidemiological features related to dental trauma.

## 5. Conclusion

This study indicated that dental trauma was very common among school children. However, few with dental trauma received dental treatment. Immediate care of dental trauma was uncommon among children. Many children with dental trauma used self-care at home and visited a private dental clinic. The most common reasons for dental trauma were the fall on the ground and accidentally hit by some objects. Monthly family income was significantly associated with dental trauma and the receipt of dental treatment. Collaborative preventive measures should be taken at the community level to reduce the burden of dental trauma and promote necessary dental care after trauma.

## Figures and Tables

**Table 1:** Demographic profile of study participants.

Study variables	Frequency (%), *N* = 258
*Class/grade*	
7^th^	90 (34.9)
8^th^	61 (23.6)
9^th^	107 (41.5)

*Monthly family income*	
Low (less than 10,000 SAR)	13 (5.0)
Middle (10,000–20,000 SAR)	132 (51.2)
High (more than 20,000 SAR)	113 (43.8)

*Paternal education*	
No school education	118 (45.7)
School education	70 (27.1)
College/university education	70 (27.1)

Academic score	
Poor (<70% grades)	10 (3.9)
Average (70%–89% grades)	47 (18.2)
Good (≥90% grades)	201 (77.9)

*Experienced dental trauma in the past*	
Yes	102 (39.5)
No	156 (60.5)
*Treatment received*	
Yes	53 (20.5)
No	205 (79.5)

*Age*	Mean ± SD
	14.29 ± 1.11

**Table 2:** Association between sociodemographic factors and dental trauma.

Variables	Unadjusted odds ratio (95% CI)	*P* value	Adjusted odds ratio (95% CI)	*P* value
*Class/grade*				
7^th^	1.69 (1.01, 2.85)	0.047	1.33 (0.72, 2.46)	0.361
8^th^	0.62 (0.34, 1.15)	0.125	0.58 (0.28, 1.2)	0.145
9^th^^*∗*^				

*Family income*				
Low	1.84 (0.6, 5.65)	0.279	0.80 (0.21, 3.07)	0.746
Middle	0.48 (0.28, 0.8)	0.004	0.44 (0.24, 0.81)	0.008
High^*∗*^				

*Paternal education*				
No school education	1.33 (0.81, 2.19)	0.266	0.99 (0.51, 1.91)	0.983
School education	0.67 (0.38, 1.2)2	0.181	0.73 (0.35, 1.54)	0.41
College/university education^*∗*^				

*Academic score*				
Poor^*∗*^				
Average	1.44 (0.76, 2.72)	0.259	2.27 (0.45, 11.43)	0.322
Good	0.79 (0.44, 1.44)	0.449	1.19 (0.26, 5.39)	0.820

^*∗*^Reference category.

**Table 3:** Association between sociodemographic factors and receipt of treatment for dental trauma.

Variables	Unadjusted odds ratio (95% CI)	*P* value	Adjusted odds ratio (95% CI)	*P* value
*Class/grade*				
7^th^	1.74 (0.94, 3.22)	0.075	1.3 (0.63, 2.66)	0.472
8^th^	0.43 (0.18, 1.10)	0.045	0.43 (0.16, 1.16)	0.096
9^th^^*∗*^				

*Family income*				
Low	1.78 (0.53, 6.01)	0.349	0.4 (0.07, 2.23)	0.297
Middle	0.41 (0.22, 0.77)	0.005	0.41 (0.2, 0.85)	0.017
High^*∗*^				

*Paternal education*				
No school education	1.18 (0.65, 2.17)	0.586	0.87 (0.4, 1.9)	0.734
School education	0.74 (0.36, 1.51)	0.409	0.8 (0.33, 1.95)	0.624
College/university education^*∗*^				

*Academic score*				
Poor^*∗*^			0.62 (0.09, 4.17)	0.620
Average	0.76 (0.33, 1.73)	0.045	0.81 (0.14, 4.51)	0.808
Good	1.28 (0.60, 2.74)	0.526		

^*∗*^Reference category.

## Data Availability

The SPSS data file of this study is available from the corresponding author upon request.
